# Treatments to Avoid Ranula Recurrence: A Network Meta‐Analysis

**DOI:** 10.1111/jop.70041

**Published:** 2025-09-08

**Authors:** Marina Rocha Fonseca Souza, Moisés Willian Aparecido Gonçalves, Roberta Rayra Martins‐Chaves, Rachel Alvarenga‐Brant, Bruno Chrcanovic, Long Ge, Honghao Lai, Ricardo Santiago Gomez, Carolina Castro Martins‐Pfeifer

**Affiliations:** ^1^ Department of Clinical Dentistry, Pathology and Oral Surgery, Faculty of Dentistry Universidade Federal de Minas Gerais Belo Horizonte Brazil; ^2^ Department of Oral Diagnosis, Piracicaba Dental School State University of Campinas (UNICAMP), Piracicaba, São Paulo, Brazil; Department of Pathology, School of Medical Sciences, State University of Campinas (UNICAMP) São Paulo Brazil; ^3^ Faculty of Medical Sciences of Minas Gerais; ^4^ Department of Oral and Maxillofacial Surgery and Oral Medicine, Faculty of Odontology Malmö University Malmö Sweden; ^5^ Evidence‐Based Social Science Research Center, School of Public Health Lanzhou University Lanzhou China; ^6^ Department of Pediatric Dentistry Universidade Federal de Minas Gerais Belo Horizonte Brazil

**Keywords:** clinical trials, meta‐analysis, ranula, recurrence, treatments

## Abstract

**Background:**

Oral and plunging ranulas require effective treatment strategies to minimize recurrence; yet no consensus exists on the most effective approach.

**Objectives:**

This systematic review evaluated several treatments for the recurrence of oral and plunging ranulas.

**Methodology:**

A comprehensive search was conducted in five bibliographic databases and gray literature. Randomized and non‐randomized studies were included if they investigated treatment approaches for oral or plunging ranulas. Two independent reviewers screened studies, extracted data, and assessed the risk of bias. The primary outcome was recurrence of (1) oral and (2) plunging ranula. For each type of ranula, a random‐model frequentist network meta‐analysis (NMA) was established for seven treatment strategies: enucleation, micromarsupialization, marsupialization, marsupialization with packing, partial sublingual gland excision, sublingual gland excision, and sublingual gland excision plus submandibular gland excision. A minimal important difference (MID) and the GRADE approach for NMA were used for interpretation of data.

**Results:**

Eighteen studies were included (all non‐randomized—14 for oral ranula and six for plunging ranula). No treatment demonstrated clear superiority in preventing recurrence. Certainty of evidence was low to very low for oral ranulas and very low for plunging ranulas, primarily due to the risk of bias, imprecision, and intransitivity.

**Conclusions:**

Given the low certainty of evidence, no single treatment can be considered superior to others. Future research should prioritize longer follow‐up randomized controlled trials.

## 1 Introduction

Ranula is a pseudocyst derived from mucus retention or salivary extravasation [1, 2]. It develops due to trauma, congenital malformation, or obstruction of the submandibular gland ducts, minor salivary glands, or the sublingual gland duct. Among these, the sublingual gland duct is the most affected by salivary leaks, as it is prone to trauma in the region, has a tortuous anatomical course, and saliva has higher viscosity compared to other glands [3, 4, 5].

Usually, the lesion is intraoral and unilateral, painless, bluish, transparent, and a floating mass on the floor of the mouth [6, 7]. Ranulas are classified as simple (oral) or plunging. Oral ranula occurs more frequently between the first and second decades of life and is limited to the submucosa of the mouth floor [8]. Plunging ranula occurs when there is a dehiscence of the mylohyoid muscle, and the sublingual gland invades the submandibular space, leading to extravasation into the neck [9]. The diagnosis can be based on clinical features; though there are other methods, such as fine needle aspiration and imaging techniques that aid in measuring the size of the lesion, such as magnetic resonance imaging (MRI) or computed tomography (CT) [7].

There are several treatments available for managing ranula: marsupialization, incision and drainage, aspiration of cystic fluid, enucleation, and excision of the sublingual or submandibular glands [1, 10, 11, 12]. The most frequently employed methods are marsupialization, excision of the sublingual gland, and excision of the ranula [13, 14]. The former involves creating a slit in the cyst wall and suturing the edges of the lesion to allow continuous drainage; it was considered the first choice for ranula treatment. However, the recurrence in larger lesions prompted the use of excision of the sublingual gland, considered a reasonable and radical treatment for ranula [13, 14]. Although the resection of the submandibular gland treatment is associated with the least risk of recurrence, this procedure is invasive, requires general anesthesia, and may result in complications, including nerve injury, damage to Wharton's duct, and bleeding [15].

Understanding recurrence and complications resulting from ranula management is important, as it can guide the selection of the most appropriate treatment for oral and plunging ranulas. A previous study found that intraoral resection of the sublingual salivary gland had the highest cure rate (98%) for the treatment of oral ranulas compared to excision of the ranula alone (89%) [15]. Though the study presented a proportion of cure rates for treatments, the aim was not to compare the treatments' effectiveness against each other. The question of the best treatment to avoid recurrence is still unanswered. Therefore, this systematic review aimed to evaluate the effectiveness of several treatments for the minimal recurrence of oral and plunging ranulas.

## 2 Materials and Methods

### 2.1 Protocol and Register

This systematic review was reported following the Preferred Reporting Items for Systematic Reviews and Meta‐analyses (PRISMA) [16]. The protocol was registered in PROSPERO (CRD42022320967). Some adjustments were made to the protocol following the commencement of the review. We modified the eligibility criteria by excluding case series and requiring a minimum of five patients per group; we added to the inclusion criteria controlled studies (minimum of two groups); and we replaced the risk of bias tool with ROBINS‐I. Additional minor changes were detailing the treatments that could be found, such as marsupialization, enucleation, partial removal of the sublingual or submandibular glands, total removal of the sublingual or submandibular glands, and others.

### 2.2 Eligibility Criteria

The clinical question was: “Is there a difference in the recurrence between marsupialization, enucleation, partial removal of sublingual or submandibular glands, total removal of sublingual or submandibular glands, and others for the treatment of ranula?”

The PICO question was (population, intervention, comparator, outcome):

(P) healthy patients at any age who were treated for oral ranula or plunging ranula;

(I) marsupialization, enucleation, partial removal of sublingual or submandibular glands, total removal of sublingual or submandibular glands, and others;

(C) the conventional marsupialization was considered the reference for comparison treatment;

(O) incidence of lesion recurrence after treatment.

#### 2.2.1 Inclusion Criteria

The inclusion criteria were clinical studies (randomized clinical trials, prospective or retrospective non‐randomized studies) reporting different treatment option details that were directly related to ranula.

#### 2.2.2 Exclusion Criteria

Studies that did not report the recurrence rate; studies that did not expressly report the type of surgical treatments or ranula classification; unavailable full texts, review papers, conference abstracts, letters to the editor; studies with unclear or incomplete data.

### 2.3 Information Sources

The following electronic databases were searched: MedLine (via PubMed), Cochrane Central Registry of Controlled Trials (CENTRAL), Virtual Health Library (VHL), Web of Science, and ScienceDirect. The search strategy in each database was performed up to March 25, 2022; it was updated on June 17, 2024. There were no restrictions on language or publication date. In addition, gray literature was verified using Google Scholar and OpenGrey. References lists of the included articles were also manually searched to identify additional relevant studies. The Clinical Trials, International Clinical Trials Registry Platform (ICTRP) were searched for clinical trials. The search strategies for each database are shown in Table S1. The retrieved studies were organized on EndNote version 20.0.1 (Clarivate Analytics, Philadelphia, PA, USA) and duplicates were excluded. Full texts in a language not mastered by the authors were translated using the Google Translate tool [17].

### 2.4 Study Selection

Two reviewers (MRFS and MWAG) independently screened titles and abstracts, and then full texts. Disagreements between reviewers regarding the included studies were resolved through discussion and consensus. If a consensus was not reached, a third reviewer (RSG) made the final decision.

### 2.5 Data Extraction

Data were extracted independently by two reviewers (MRFS and MWAG) using a data extraction form in Microsoft Excel 2016 (Microsoft, Redmond, WA, USA) file format: author, year of publication, country, eligibility criteria, sample size, study design, follow‐up period, intervention details, patients' characteristics (sex and age), type of ranula, sample per group, number of patients with recurrence, adverse effects, funding agency.

### 2.6 Risk of Bias

Paired independent reviewers (MRFS and MWAG) assessed the studies' risk of bias following the ROBINS‐I tool for non‐randomized studies of interventions [18]. The senior author (CCM‐P) trained the reviewers using 10% of the trials. Disagreements were resolved by consensus between the pair of reviewers.

### 2.7 Data Synthesis and Statistical Methods

The primary outcome was recurrence, which was treated separately for oral and plunging ranula, as described below. The secondary outcome was adverse effects.

A frequency distribution was calculated for study characteristics using Microsoft Excel. Two random‐effect model frequentist NMA were run for oral and plunging ranula, including seven intervention groups: micromarsupialization, partial sublingual gland excision, enucleation, sublingual gland excision plus submandibular gland excision, marsupialization, marsupialization with packing, and sublingual gland excision. As many studies reported a recurrence equal to zero for some treatments, the model used a strategy of adding a continuity correction of 0.5 to all cells, which allowed the inclusion of all studies in the network [19]. Therefore, the risk ratio (RR) and 95% confidence intervals (CI) comparing two treatments were calculated. Incoherence (i.e., inconsistency in the model) was assessed by comparing direct estimates with indirect estimates and final network estimates using the back‐calculation method. Incoherence in the entire network was evaluated via a design‐by‐treatment model with a 2‐tailed threshold of *p* ≤ 0.05, indicating statistically significant incoherence [20]. All analyses were conducted using R version 3.4.3 (R Core Team) with the *netmeta* package version 1.4‐0.

### 2.8 Certainty of Evidence

The Grading of Recommendation Assessment, Development, and Evaluation (GRADE) tool was used to assess the certainty of the evidence. The evaluation started from high‐quality evidence when the risk of bias was assessed using ROBINS‐I [21]. The direct evidence was rated down if there were problems due to risk of bias, inconsistency, indirectness, and publication bias. The indirect evidence was rated down if there were problems due to intransitivity. Finally, the network estimate was rated down if there were problems due to imprecision and incoherence [22, 23]. The detailed approach is described in Table S2.

The minimal important difference (MID) was considered for a treatment decision comparing intervention and comparator [23, 24, 25]. The MID threshold was set at 10% for oral and plunging ranula, which means an RR of 0.9 (less recurrence compared to the other treatment) and 1.1 (more recurrence compared to the other treatment). More details are in Table S2.

## 3 Results

### 3.1 Study Selection

**FIGURE 1:**
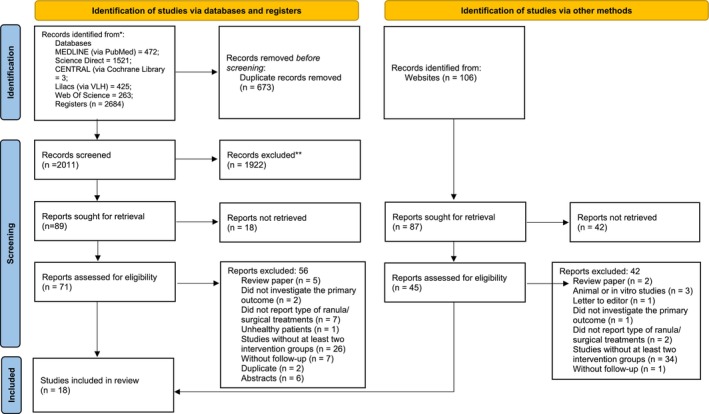
PRISMA flow diagram.

The search strategy retrieved 2684 articles. After the screening process, the review included 18 non‐randomized studies of intervention: 14 contributed to the NMA for oral ranula and six for plunging ranula. The study selection process is summarized in Figure 1. Appendix S1: Reference 1 shows the list of included studies, and Table S3 lists the excluded studies with reasons.

### 3.2 Studies Characteristics

Table 1 presents the study characteristics. Most studies were conducted in Asia (50%) and published in the English language (100%) and after 2000 (89%). No study was industry funded. The total number of patients for oral ranula was 413, and for plunging ranula was 97, with patients' age ranging from 2 days to 59 years.

**TABLE 1:** Summary of study characteristics.

Characteristic	Number of non‐randomized studies of interventions 18 (100%)
Language	
English	18 (100%)
Continents (authors from)	
Asia^a^	9 (50%)
Europe^b^	3 (16%)
North America^c^	2 (11%)
South America^d^	2 (11%)
Oceania^e^	1 (6%)
Africa^f^	1 (6%)
Year of publication	
1990–1999	2 (11%)
2000–2009	8 (44%)
2010–2018	2 (11%)
2019–2024	6 (34%)
Setting	
Dental school/surgery clinic/hospital	17 (94%)
Research center	1 (6%)
Funding	
Not reported	12 (67%)
Government/University funding	2 (11%)
No funding	4 (22%)
Conflict of Interests	
No	9 (50%)
Not reported	9 (50%)
Final sample	
Oral Ranula	
Minimum	1
Maximum	68
Total	413
Plunging Ranula	
Minimum	1
Maximum	22
Total	97
Age of patients	
Minimum	2 days
Maximum	59 years
% of women	43.6%
Follow up	
Minimum	6 weeks
Maximum	364 weeks

^a^ Turkey, China, India, Japan, South Korea, Republic of Korea.

^b^ Italy, Poland, Belgium.

^c^ USA.

^d^ Brazil.

^e^ New Zealand.

^f^ Nigeria.

### 3.3 Risk of Bias

**FIGURE 2:**
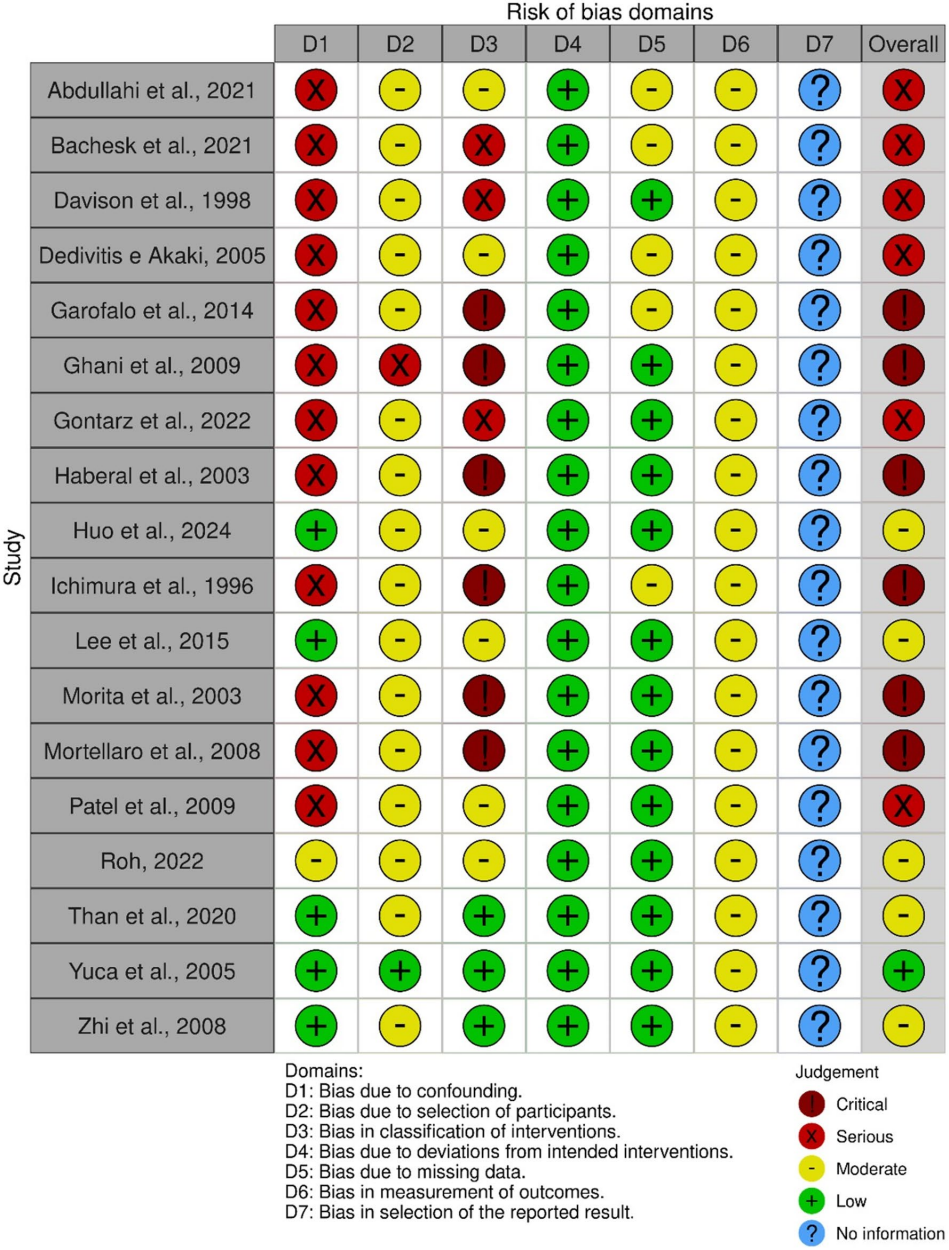
Risk of bias of 18 studies using ROBINS‐I. Critical risk of bias represented in dark red; high risk of bias represented in red; some concerns represented in orange; low risk of bias represented in green.

Overall, 5.5% of studies were at low risk of bias, 27.7% were at moderate risk of bias, 33.3% were at high risk of bias, and 33.3% were at critical risk of bias (Figure 2). The studies were judged at low risk of bias regarding “confounding” (27.7%), “selection of participants” (5.5%), “classification of interventions” (16.6%), “deviations from intended interventions” (100.0%), “missing data” (72.2%). However, all studies had some concerns regarding “measurement of the outcomes” (100%), and no study presented information about “selection of the reported result”.

### 3.4 Recurrence

**FIGURE 3:**
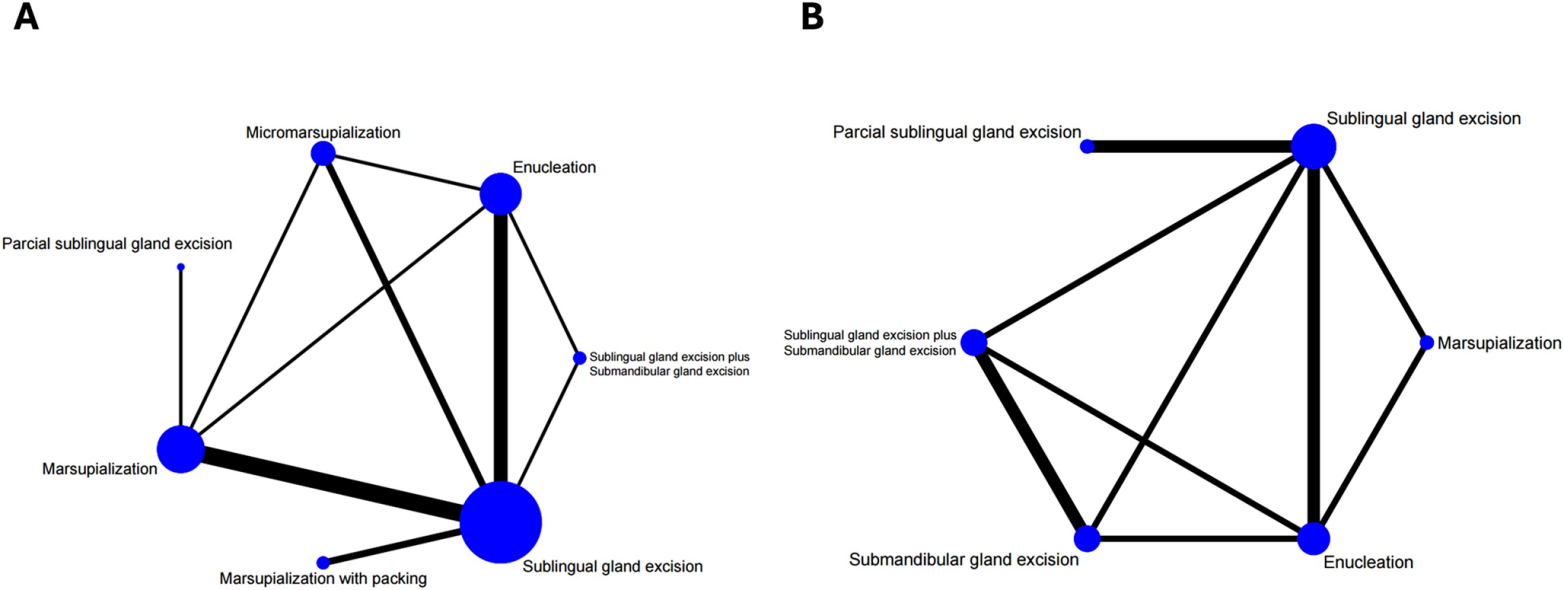
(A) Network geometry for recurrence—oral ranul. (B) Network geometry for recurrence—plunging ranula. The thickness of continuous lines represents the number of studies, in order that the thicker lines have more studies in the comparison. In the same way, the bigger balls have more patients in the treatment arm.

The network geometries are represented in Figure 3A,B for oral and plunging ranula. Fourteen studies were included for oral ranula, and 6 studies for plunging ranula. For oral ranula, all treatment comparisons had low to very low certainty, which shows the lack of certainty regarding their recurrence (Figure 3A, Table 2). Recurrence was higher for marsupialization with packing than for micromarsupialization (RR: 11.69; 95% CI: 1.19–114.66, low certainty), though based on indirect evidence. All treatment comparisons had very low certainty of the evidence for plunging ranula (Figure 3B, Table 3).

**TABLE 2:** Oral ranula recurrence, random‐effect model.

Enucleation	0.88 (0.21; 3.77)	.	3.57 (0.27; 46.49)	.	0.18 (0.01; 5.93)	1.58 (0.59; 4.21)
0.98 (0.33; 2.91)^d^	Marsupialization	.	1.80 (0.15; 21.48)	0.33 (0.01; 12.82)	.	1.92 (0.50; 7.41)
0.26 (0.03; 2.20)^d^, ^e^	0.27 (0.03; 2.42)^d^, ^e^	Marsupialization with packing	.	.	.	5.09 (0.74; 34.95)
3.09 (0.78; 12.24)^b^, ^d^	3.16 (0.77; 12.97)^a^, ^c^	11.69 (1.19; 114.66)^c^, ^e^	Micromarsupialization	.	.	0.36 (0.09; 1.42)
0.33 (0.01; 14.70)^d^, ^e^	0.33 (0.01; 12.82)^d^	1.23 (0.02; 6.94) ^d,e^	0.11 (0.00; 5.28)^d^, ^e^	Partial sublingual gland excision	.	.
0.58 (0.04; 8.45)^a^, ^d^	0.59 (0.04; 9.46)^d^, ^e^	2.18 (0.09; 55.48) ^d,e^	0.19 (0.01; 3.26)^d^, ^e^	1.77 (0.02; 173.47)^d^, ^e^	Sublingual gland excision plus Submandibular gland excision	1.67 (0.11; 24.26)
1.35 (0.56; 3.26)^a,d^	1.38 (0.49; 3.91)^a,d^	5.09 (0.74; 34.95)^b,d^	0.44 (0.13; 1.49)^a^, ^d^	4.13 (0.09; 183.98)^d^, ^e^	2.33 (0.17; 1.39)^a^, ^d^	Sublingual gland excision

*Note*: The lower table shows the network estimates; the upper table shows the direct estimates. The effectiveness estimate is located at the intersection of the column‐defining treatment and the row‐defining treatment (black cells). Risk ratio (RR) and respective 95% CI represent the recurrence of oral ranula after treatment. Values above 1 favor treatment on the column (more recurrence for the treatment on the column), and values below 1 favor treatment on the row (more recurrence for the treatment on the row). Cells' colors represent the certainty of the evidence, as the following interpretation: High certainty (not represented in the chart): further research is unlikely to change the confidence in the effect estimate. Moderate certainty (not represented in the chart): further research is likely to have an important impact on the confidence in the effect estimate and may change the estimate. Low (medium gray): further research is very likely to have an important impact on the confidence in the effect estimate and is likely to change the estimate. Very low (light gray): any estimate of effect is very uncertain.

^a^ Serious risk of bias.

^b^ Very serious risk of bias.

^c^ Serious imprecision.

^d^ Very serious imprecision.

^e^ Serious intransitivity.

**TABLE 3:** Plunging ranula recurrence, random‐effect model.

Enucleation	1.19 (0.36; 3.99)	.	2.33 (0.16; 34.89)	1.00 (0.04; 24.55)	5.95 (0.91; 38.92)
1.19 (0.36; 3.95)^a^, ^b^	Marsupialization	.	.	.	3.00 (0.21; 43.66)
0.54 (0.03; 8.62)^b^, ^c^	0.45 (0.02; 8.78)^b^, ^c^	Partial sublingual gland excision	.	.	6.33 (0.68; 58.96)
5.37 (0.67; 43.13)^a^, ^b^	4.52 (0.43; 48.08)^b^, ^c^	9.95 (0.49; 200.29)^b^, ^c^	Sublingual gland excision plus Submandibular gland excision	0.77 (0.09; 6.72)	0.47 (0.03; 7.36)
4.68 (0.45; 48.87)^a^, ^b^	3.94 (0.29; 52.71)^b^, ^c^	8.67 (0.38; 198.60)^b^, ^c^	0.87 (0.10; 7.54)^a^, ^b^	Submandibular gland excision	0.21 (0.01; 3.88)
3.41 (0.66; 17.67)^b^, ^d^	2.88 (0.41; 20.15)^b^, ^d^	6.33 (0.68; 58.96)^b^, ^d^	0.64 (0.09; 4.74)^b^, ^d^	0.73 (0.08; 6.57)^b^, ^d^	Sublingual gland excision

*Note*: The lower table shows the network estimates; the upper table shows the direct estimates. The effectiveness estimate is located at the intersection of the column‐defining treatment and the row‐defining treatment (black cells). Risk ratio (RR) and respective 95% CI represent the recurrence of plunging ranula after treatment. Values above 1 favor treatment on the column (more recurrence for the treatment on the column), and values below 1 favor treatment on the row (more recurrence for the treatment on the row). Cells' colors represent the certainty of the evidence, as the following interpretation: High certainty (not represented in the chart): further research is unlikely to change the confidence in the effect estimate. Moderate certainty (not represented in the chart): further research is likely to have an important impact on the confidence in the effect estimate and may change the estimate. Low (not represented in the chart): further research is very likely to have an important impact on the confidence in the effect estimate and is likely to change the estimate. Very low (light gray): any estimate of effect is very uncertain.

^a^ Very serious risk of bias.

^b^ Very serious imprecision.

^c^ Serious intransitivity.

^d^ Serious risk of bias.

Many treatments did not achieve the MID, which means the minimal benefit to justify the use of one in detriment to another treatment. The MID was set at 0.9 or 1.1 (which means a maximum of 10% of recurrence, Table S2). Even though other treatments achieved the MID, the 95% CI crossed either the superior or the inferior thresholds and the null effect line. That being said, many network estimates were rated down by two levels due to very serious imprecision. It means that either the reference treatment or the comparison could increase recurrence (imprecise estimates). Many other estimates were rated down due to serious or very serious risk of bias. In summary, no treatment was superior to the other; therefore, no ranking probability was created for this study. The direct and network estimates are detailed in the Appendix S1: Plots 1–4.

## 4 Discussion

Though a reasonable number of treatments showed positive results, no treatment demonstrated clear superiority in preventing recurrence for both oral and plunging ranulas. Therefore, this discussion centers on the lack of definitive conclusions regarding the most effective treatment based on the available data observed from the NMA.

The lack of clear superiority between treatments aligns with the variability observed in the literature, where studies report mixed success rates depending on various factors such as surgical technique, professional experience, extent of gland removal, and follow‐up duration [15]. The recurrence is primarily driven by persistent trauma or dysfunction of the affected salivary gland [8]. If the source of mucus leakage remains active—whether due to incomplete gland removal, continued ductal obstruction/rupture, or repetitive mechanical irritation—fluid accumulation can recur regardless of the treatment performed. This highlights the importance of addressing the cause rather than merely managing the lesion.

A wide range of treatments is proposed for the management of oral ranulas. Among these, marsupialization and its variations, including marsupialization with packing, are frequently used. Marsupialization relies on the presence of a true cyst lining to maintain the newly created epithelialized opening, allowing continuous drainage and preventing recurrence [26]. However, ranulas result from mucus extravasation rather than a true cystic structure, meaning their walls lack a well‐defined epithelial lining [8, 27]. Marsupialization with packing involves unroofing the pseudocystic cavity and firmly packing it with gauze rather than leaving it open [28].

Despite previous reports suggesting that marsupialization with packing significantly reduces recurrence [10, 28], it is noteworthy that no prior studies directly compared marsupialization and packing versus micromarsupialization were found in the literature. The higher recurrence of marsupialization with packing compared to micromarsupialization observed in our findings should be interpreted with caution due to several factors. First, the associations found in our data were indirect, limiting the applicability of the results to all clinical conditions [23]. The reliance on indirect estimates introduces uncertainty, justifying the downgrading of the certainty of this evidence [23]. Second, the studies analyzed involve patients with different follow‐up periods, which may influence recurrence rates, as some treatments lead to relapses at varying speeds. Third, though the large magnitude of the effect and the 95% CI did not cross the null effect line, the CI was very large, showing high imprecision. Additionally, there is no biologically plausible explanation for this result, and statistical significance without biological plausibility should be considered a secondary finding, possibly occurring by chance. Micromarsupialization is a minimally invasive technique for treating oral ranulas, requiring only a single suture to address lesions [29]. This approach has resulted in high recurrence [30, 31] and some studies have suggested it is less reliable than intraoral resection of the sublingual gland [15, 32]. In clinical practice, micromarsupialization is rarely used due to its high chance of recurrence. Finally, the low certainty indicates a great probability that the true estimate might differ greatly from the current one [33].

In general, the great limitation of the studies was the risk of biased assessment which revealed considerable variability in methodological quality. A substantial proportion of the studies (66.6%) were identified as having a high or critical risk of bias, particularly in areas such as “deviations from intended interventions” and “missing data.” While some studies demonstrated low bias in aspects like confounding factors, participant selection, and classification of interventions, all studies exhibited concerns related to the “measurement of outcomes,” highlighting limitations in the current evidence.

### 4.1 Strength and Limitations

This study has some limitations. The limited number of events in each group resulted in imprecision of most effect estimates. This might have been affected by our decision to analyze treatment options separately instead of trying to group them (which could increase the number of events in each group). However, we considered clinical interest instead of statistical interest when making this decision.

A strong point was our decision to use a continuity correction that enabled groups with recurrence equal to zero to enter the NMA [19, 20]. Another strong point is the comprehensiveness of this review, which employs a robust methodology and utilizes the MID for the effect magnitude, certainty of the evidence, and decision thresholds that were cautiously established to avoid misleading interpretations [25, 34].

### 4.2 Perspectives for Clinical Practice and Research

With the current lack of superiority among treatments, clinicians should tailor their approach based on individual patient factors, including lesion size, severity, and the presence of plunging extension. This suggests that less invasive techniques may still be viable options, particularly when surgical risks must be minimized. Future research should focus on long‐term randomized‐controlled trials to evaluate the recurrence resulting from these treatments.

## 5 Conclusion

No treatment proved superior in preventing ranula recurrence, emphasizing the need for individualized approaches.

## Author Contributions

Marina Rocha Fonseca Souza contributed to the conception, design, data acquisition, and interpretation, drafted and critically revised the manuscript. Moisés Willian Aparecido Gonçalves, Roberta Rayra Martins‐Chaves, Rachel Alvarenga‐Brant, Bruno Chrcanovic, Long Ge, Honghao Lai, Ricardo Santiago Gomez contributed to the data acquisition and interpretation, and critically revised the manuscript. Carolina Castro Martins‐Pfeifer contributed to the conception, design, and critically revised the manuscript. All authors gave their final approval and agreed to be accountable for all aspects of the work.

## Conflicts of Interest

The authors declare no conflicts of interest.

## Supporting information


**Appendix S1:** Supporting Information.

## Data Availability

The data that support the findings of this study are available upon request.
