# Supplementary material for: Treatments to Avoid Ranula Recurrence: A Network Meta‐Analysis
Source: J Oral Pathol Med. 2025 Sep 8;54(10):934–43. doi: 10.1111/jop.70041 (PMC12602136; doi:10.1111/jop.70041)
Supplement: Supplementary file 1 — Appendix S1: Supporting Information. [file JOP-54-934-s001.docx]

**TREATMENTS TO AVOID RANULA RECURRENCE:**

**A NETWORK META-ANALYSIS**

Marina Rocha Fonseca Souza^1^, Moisés Willian Aparecido Gonçalves^2^, Roberta Rayra Martins-Chaves^1^, Rachel Alvarenga-Brant^1^, Bruno Chrcanovic^3^, G. Long^4^, L. Honghao^5^, Ricardo Santiago Gomez^6^, Carolina Castro Martins-Pfeifer^7^.

**Authors affiliations:**

^1^Ph.D. student, Department of Clinical Dentistry, Pathology and Oral Surgery, Faculty of Dentistry, Federal University of Minas Gerais, Belo Horizonte, Brazil.

^2^Ph.D. student, Department of Oral Diagnosis, Piracicaba Dental School, State University of Campinas (UNICAMP), Piracicaba, São Paulo, Brazil; Department of Pathology, School of Medical Sciences, State University of Campinas (UNICAMP), Campinas, São Paulo, Brazil.

^3^ Associate Professor, Department of Oral and Maxillofacial Surgery and Oral Medicine, Faculty of Odontology, Malmö University, Malmö, Sweden.

^4^Professor, Evidence-Based Social Science Research Center, School of Public Health, Lanzhou University, Lanzhou, China.

^5^Ph.D. student, Evidence-Based Social Science Research Center, School of Public Health, Lanzhou University, Lanzhou, China.

^6^Full Professor, Department of Clinical Dentistry, Pathology and Oral Surgery, Faculty of Dentistry, Federal University of Minas Gerais, Belo Horizonte, Brazil.

^7^Associate Professor, Department of Pediatric Dentistry, Federal University of Minas Gerais, Belo Horizonte, Brazil.

**Appendix Table 1.** Search strategies used according to electronic databases (date: from inception to March 2022, updated on June 17, 2024).

| **Search*** | **Query** | **Records retrieved 2022** | **Records retrieved 2024** |
| --- | --- | --- | --- |
| MedLine (via Pubmed) | ((ranula) OR (ranulas) OR (oral ranula) OR (oral ranulas)) AND ((treatment) OR (therapeutic) OR (therapeutics) OR (therapy) OR (therapies) OR (treatments) OR (marsupialization) OR (marsupialised) OR (marsupializations) OR (marsupialize) OR (marsupialized) OR (marsupializing) OR (resect) OR (resectability) OR (resectable) OR (resectates) OR (resected) OR (resecting) OR (resection) OR (resectional) OR (resectioned) OR (resectioning) OR (resections) OR (resective) OR (resects)) | 442 | 472 |
| Lilacs  (via Virtual Health Library) | ((Rânula) OR (Ranula) OR (Ránula)) AND ((Ação Terapêutica) OR (Ações Terapêuticas) OR (Medida Terapêutica) OR (Medidas Terapêuticas) OR (Procedimento Curativo) OR (Procedimento Terapêutico) OR (Procedimento de Terapia) OR (Procedimento de Tratamento) OR (Procedimentos Curativos) OR (Procedimentos Terapêuticos) OR (Procedimentos de Terapia) OR (Procedimentos de Tratamento) OR (Propriedade Terapêutica) OR (Terapia) OR  (Terapias) OR (Tratamento) OR (Tratamentos)) | 396 | 425 |
| CENTRAL (via Cochrane Library) | (Ranula) OR (Ranulas) in Title Abstract Keyword AND (Treatments) OR (Treatment) OR (Therapy) OR (Therapeutic) OR (Therapies) in Title Abstract Keyword | 03 | 03 |
| Web of science | (ALL=(ranula* OR ranulas)) AND ALL=(treatment* OR therapeutic* OR therapy* OR therapies* OR treatments) | 233 | 263 |
| Science Direct | (ranula OR ranulas) AND (treatments OR treatment OR therapy OR marsupialization OR marsupialised OR resect) | 1504 | 1521 |
| Clinical Trials | <https://clinicaltrials.gov/>  Treatment for Ranula and (recurrence) | 0 | 0 |
| The WHO International Clinical Trials Registry Plataform (ICTRP) | <https://www.who.int/ictrp/en/>  Treatment for Ranula and (recurrence) | 0 | 0 |
| Total |  | 2578 | 2684 |

*Search terms were used as MeSH descriptors and/or as title and abstract keywords, as appropriate.

**Appendix Table 2**. Description of criteria used to assess the certainty of evidence.

|  | **Rated down direct estimate if:** |
| --- | --- |
| **Risk of bias** | The risk of bias was rated down if one or more studies had an overall critical or high risk of bias [1]. |
| **Inconsistency** | - If effect estimates varied across studies [2]; - Lack of overlap of 95%CI [2]; - *I^2^* for direct comparisons was either moderate (30-60%), substantial (50-90%) or considerable (75%-100%) [3]. - When a single study was included in a comparison, the inconsistency was not rated down [2]. |
| **Indirectness** | Indirectness was assessed considering the applicability of intervention according to the clinical question (patient, interventions, outcome) [4–6]. For this study, we investigated whether the evidence would apply to all populations. Study data were from medical records, showing great variability of age groups and sex that would represent the target population. |
| **Publication bias** | Publication bias could be downgraded if:  More than 70% of the weight of the pooled effect estimate comes from studies funded by the industry for which the pooled estimate shows favorable evidence [1];  Asymmetrical funnel plot;  Significant p-value by Egger test (p< 0.05) [2].  The NMAs had no industry-funded studies, funnel plot asymmetry, or significant p-value to justify downgrading for publication bias. |
|  | **Rated down indirect estimate if:** |
| **Intransitivity** | For intransitivity, we assessed the most dominant first-order loop and considered the criteria for indirectness described above. If the evidence coming from the two direct comparisons of the loop could modify the treatment effect that formed the indirect estimate of the loop, the certainty was downgraded due to intransitivity[5]. We judged that there was no reason to downgrade due to intransitivity.  Moreover, intransitivity was rated down by one level if the effect estimate was based only on the indirect estimate [5]. |
|  | **Rated down NMA estimate if:** |
| **Incoherence** | Incoherence was assessed by comparing direct estimates, indirect estimates, and the network estimate using the back-calculation method [6]. Whenever the p-value was >0.05, the incoherence was not serious, and incoherence was not rated down. If the direct and network estimates pointed out in the same direction, the certainty was not rated down even if p<0.05. The certainty of the evidence was rated down if p<0.05, and the direct estimate pointed out in the opposite direction of the indirect estimate and the network estimate [5]. |
| **Imprecision** | For imprecision, we considered the minimum important difference (MID) required for a treatment decision comparing intervention and comparator [7]. If the 95% CI crossed the MID decision threshold, the certainty of the evidence was rated down by one level; and by two levels if the 95% CI also crossed the null effect line. If the intervention effect estimate was greater or less than the MID, the intervention was considered protective or promotive compared to the control, according to the direction of the effect estimate [4].  For MID, we considered the recurrent rate in the total population that entered the NMA from our data. There were 33 patients with recurrent oral ranula in a sample of 392. The following formula was used to calculate the rate: number of patients with recurrent ranula/ total sample*100. For oral ranula, the calculation was: 33/392*100 = 8%. There were 12 patients with recurrent plunging ranula in 88 (12/88*100= 13%). Therefore, we set a recurrence rate of a maximum of 10% for both the thresholds for oral and plunging ranula, which was set at 0.9 and 1.1. Values ​​greater than 1 indicate that the intervention had more recurrence when compared to the control; values ​​<1 indicate that the intervention had less recurrence compared to the control. |

**Appendix References 1.** List of included studies in the systematic review.

Abdullahi M, Bachesk AB, Bin LR, Iwaki IV, Iwaki Filho L, Davison MJ, et al. Ranula: A retrospective clinicosurgical analysis of 29 cases from a tertiary health institution, Northwest, Nigeria. J Clin Sci. 2021 Apr-Jun;18(2):109.

Bachesk AB, Bin LR, Iwaki IV, Iwaki Filho L. Ranula in children: Retrospective study of 25 years and literature review of the plunging variable. Int J Pediatr Otorhinolaryngol. 2021 Sep;148:110810.

Davison MJ, Morton RP, McIvor NP. Plunging ranula: clinical observations. Head Neck. 1998 Jan;20(1):63-8.

Dedivitis RA, Akaki LF. Tratamento cirúrgico da rânula. Aq Med ABC. 2005;30(2):87-9.

Garofalo S, Mussa A, Mostert M, et al. Successful medical treatment for ranula in children. Oral Surg Oral Med Oral Pathol Oral Radiol. 2014;117

Ghani NA, Ahmad R, Rahman RA, Yunus MR, Putra SP, Ramli R. A retrospective study of ranula in two centres in Malaysia. J Maxillofac Oral Surg. 2009 Dec;8(4):316-9.

Gontarz M, Bargiel J, Gąsiorowski K, Marecik T, Szczurowski P, Zapała J, et al. Surgical treatment of sublingual gland ranulas. Int Arch Otorhinolaryngol. 2022 Dec;27(2)

Haberal I, Göçmen H, Samim E. Surgical management of pediatric ranula. Int J Pediatr Otorhinolaryngol. 2004 Feb;68(2):161-3.

Huo BB. Retrospective comparative clinical study on clinical effect of suture micromarsupialization on ranula. Acta Odontol Scand. 2024 May 3;83:249-254.Ichimura K, Ohta Y, Tayama N. Surgical management of the plunging ranula: a review of seven cases. J Laryngol Otol. 1996 Jun;110(6):554-6.

Lee DH, Yoon TM, Lee JK, Lim SC. Treatment outcomes of the intraoral approach for a simple ranula. Oral Surg Oral Med Oral Pathol Oral Radiol. 2015 Apr;119(4)

Morita Y, Sato K, Kawana M, Takahasi S, Ikarashi F. Treatment of ranula--excision of the sublingual gland versus marsupialization. Auris Nasus Larynx. 2003 Aug;30(3):311-4.

Mortellaro C, Dall'Oca S, Lucchina AG, Castiglia A, Farronato G, Fenini E, et al. Sublingual ranula: a closer look to its surgical management. J Craniofac Surg. 2008 Jan;19(1):286-90.

Patel MR, Deal AM, Shockley WW. Oral and plunging ranulas: What is the most effective treatment? Laryngoscope. 2009 Aug;119(8):1501-9.

Roh JL. Transoral complete vs partial excision of the sublingual gland for plunging ranula. Otolaryngol Head Neck Surg. 2022 Sep;167(3):479-83.

Than JK, Rosenberg TL, Anand G, Sitton M. The importance of sublingual gland removal in treatment of ranulas: a large retrospective study. Am J Otolaryngol. 2020 May-Jun;41(3):102418.

Yuca K, Bayram I, Cankaya H, Caksen H, Kiroğlu AF, Kiriş M. Pediatric intraoral ranulas: an analysis of nine cases. Tohoku J Exp Med. 2005 Feb;205(2):151-5.

Zhi K, Wen Y, Ren W, Zhang Y. Management of infant ranula. Int J Pediatr Otorhinolaryngol. 2008;72:823-6.

**Appendix Table 3.** Studies excluded after full-text analysis and reasons for exclusion.

| **References** | **Reason for exclusion** |
| --- | --- |
| Abdul-Aziz D, Adil E. Ranula excision. *Operative Techniques in Otolaryngology-Head and Neck Surgery*. 2015;26(1):21-27. doi:10.1016/j.otot.2015.01.005 | Review paper. |
| Madeira AA. Contribuição para o estudo da utilização da técnica cirúrgica de enucleação total de rânulas, em indivíduos de ambos os sexos, residentes em Santa Catarina [dissertação]. Florianópolis: Universidade Federal de Santa Catarina; 1974. Disponível em: https://repositorio.ufsc.br/handle/123456789/112216 | The study did not report the type of ranula/ surgical treatments. |
| Aluko-Olokun B, Olaitan AA. Ranula Decompression Using Stitch and Stab Method: The Aluko Technique. J Maxillofac Oral Surg. 2017 Jun;16(2):192-196. doi: 10.1007/s12663-016-0971-x. Epub 2016 Sep 22. PMID: 28439160; PMCID: PMC5385694. | Study without a comparison group. |
| Amaral MBF. Tratamento do fenômeno de extravasamento/retenção de muco pela técnica de micro-marsupialização modificada associado ou não ao uso do laser de baixa intensidade: ensaio clínico controlado e randomizado [dissertação]. Belo Horizonte: Universidade Federal de Minas Gerais; 2011. Orientador: Mesquita RA. | Study without a comparison group. |
| Amaral MB, de Freitas JB, Mesquita RA. Upgrading of the micro-marsupialisation technique for the management of mucus extravasation or retention phenomena. Int J Oral Maxillofac Surg. 2012 Dec;41(12):1527-31. doi: 10.1016/j.ijom.2012.04.016. Epub 2012 May 19. PMID: 22613811. | Study without a comparison group. |
| ASHERSON N, RUNDLE WJ. Ranula: (cysts in the floor of the mouth) treatment by radiation. J Laryngol Otol. 1960 Jan;74:52-8. doi: 10.1017/s0022215100056218. PMID: 13794734. | Study without a comparison group. |
| Balakrishnan A, Ford GR, Bailey CM. Plunging ranula following bilateral submandibular duct transposition. J Laryngol Otol. 1991 Aug;105(8):667-9. doi: 10.1017/s0022215100116986. PMID: 1919326. | Study without a comparison group. |
| Barreda M, et al. Complication prevalence in total resection of sublingual glands in ranulas: experience in a service of maxillofacial in Chile. *Int J Oral Maxillofac Surg.* 2011;40(10):1171. | Abstract. |
| Baurmash HD. Marsupialization for treatment of oral ranula: a second look at the procedure. J Oral Maxillofac Surg. 1992 Dec;50(12):1274-9. doi: 10.1016/0278-2391(92)90226-p. PMID: 1447605. | Study without a comparison group. |
| Black RJ, Croft CB. Ranula: pathogenesis and management. Clin Otolaryngol Allied Sci. 1982 Oct;7(5):299-303. doi: 10.1111/j.1365-2273.1982.tb01910.x. PMID: 7172464. | The study did not investigate the primary outcome. |
| Bonet-Coloma C, Minguez-Martinez I, Aloy-Prósper A, Galán-Gil S, Peñarrocha-Diago M, Mínguez-Sanz JM. Pediatric oral ranula: clinical follow-up study of 57 cases. Med Oral Patol Oral Cir Bucal. 2011 Mar 1;16(2):e158-62. doi: 10.4317/medoral.16.e158. PMID: 21196880. | Study without a comparison group. |
| Bowers EMR, Schaitkin B. Management of Mucoceles, Sialoceles, and Ranulas. Otolaryngol Clin North Am. 2021 Jun;54(3):543-551. doi: 10.1016/j.otc.2021.03.002. PMID: 34024482. | Review paper. |
| Chen JX, Zenga J, Emerick K, Deschler D. Sublingual gland excision for the surgical management of plunging ranula. Am J Otolaryngol. 2018 Sep-Oct;39(5):497-500. doi: 10.1016/j.amjoto.2018.05.011. Epub 2018 May 26. PMID: 30017374. | Study without a comparison group. |
| Chidzonga MM, Mahomva L. Ranula: experience with 83 cases in Zimbabwe. J Oral Maxillofac Surg. 2007 Jan;65(1):79-82. doi: 10.1016/j.joms.2005.10.058. PMID: 17174768. | Unhealthy patients. |
| Chung IK, Lee HJ, Hwang DS, Kim YD, Park HR, Shin SH, Kim UK, Lee JY. Partial sublingual glandectomy with ranula excision: a new conservative method for treatment. JKAOMS 2012;38:160-165. https://doi.org/10.5125/jkaoms.2012.38.3.160 | The study did not report the type of ranula/ surgical treatments. |
| Crysdale WS, Mendelsohn JD, Conley S. Ranulas-mucoceles of the oral cavity: experience in 26 children. Laryngoscope. 1988 Mar;98(3):296-8. doi: 10.1288/00005537-198803000-00011. PMID: 3343879. | The study did not report the type of ranula/ surgical treatments. |
| George K. Surgical techniques for parotid and submandibular glands and ranulae. *Maxillofacial Surgery*. 2017. p. 686-699. doi:10.1016/B978-0-7020-6056-4.00049-6. | Study without a comparison group. |
| Baurmash HD. Mucoceles and ranulas. J Oral Maxillofac Surg. 2003 Mar;61(3):369-78. doi: 10.1053/joms.2003.50074. PMID: 12618979. | Review paper. |
| Hills A, Holden A, McGurk M. Evolution of the management of ranulas: change in a single surgeon's practice 2001-14. Br J Oral Maxillofac Surg. 2016 Nov;54(9):992-996. doi: 10.1016/j.bjoms.2016.07.005. Epub 2016 Jul 25. PMID: 27460783. | Study without a comparison group. |
| Huang SF, Liao CT, Chin SC, Chen IH. Transoral approach for plunging ranula--10-year experience. Laryngoscope. 2010 Jan;120(1):53-7. doi: 10.1002/lary.20674. PMID: 19877177. | Study without follow-up. |
| Jain P, Jain R, Morton RP, Ahmad Z. Plunging ranulas: high-resolution ultrasound for diagnosis and surgical management. Eur Radiol. 2010 Jun;20(6):1442-9. doi: 10.1007/s00330-009-1666-1. Epub 2009 Nov 27. PMID: 19943050. | The study did not investigate the primary outcome. |
| Jain R, Morton RP, Ahmad Z. Diagnostic difficulties of plunging ranula: case series. J Laryngol Otol. 2012 May;126(5):506-10. doi: 10.1017/S0022215112000230. Epub 2012 Mar 9. PMID: 22401594. | Study without follow-up. |
| Jia, Y; Zhao, Y; Chen, X. Clinical and histopathological review of 229 cases of ranula. J HuazhongUniv Sci Technol. 2011;31(5):717-720 | Study without a comparison group. |
| Jia T, Xing L, Zhu F, Jin X, Liu L, Tao J, Chen Y, Gao Z, Zhang H. Minimally invasive treatment of oral ranula with a mucosal tunnel. Br J Oral Maxillofac Surg. 2015 Feb;53(2):138-41. doi: 10.1016/j.bjoms.2014.10.015. Epub 2014 Nov 20. PMID: 25468316. | Study without a comparison group. |
| Payne KFB, Goodson AMC, George K, McGurk M. Modified micro-marsupialisation: A novel technique for minimally invasive management of oral ranula. Oral Surg Oral Med Oral Pathol Oral Radiol. 2014 Oct;52(8):e122-e123. | Abstract. |
| KEEN P, COHEN L, SHAPIRO MP. Plunging ranula: a new therapeutic approach. S Afr Med J. 1954 Mar 6;28(10):189-93. PMID: 13146333. | Plunging ranula: a new therapeutic approach. |
| Kokong D, Iduh A, Chukwu I, Mugu J, Nuhu S, Augustine S. Ranula: Current Concept of Pathophysiologic Basis and Surgical Management Options. World J Surg. 2017 Jun;41(6):1476-1481. doi: 10.1007/s00268-017-3901-2. PMID: 28194490; PMCID: PMC5422487. | Study without a comparison group. |
| Lomas J, Chandran D, Whitfield BCS. Surgical management of plunging ranulas: a 10-year case series in South East Queensland. ANZ J Surg. 2018 Oct;88(10):1043-1046. doi: 10.1111/ans.14356. Epub 2017 Dec 21. PMID: 29266658. | Study without follow-up. |
| Lopez P. Surgical management of ranulas: A retrospective study. Oral Surg Oral Med Oral Pathol Oral Radiol. 2005 Aug;63(8 Suppl):80. | Abstract. |
| Mahadevan M, Vasan N. Management of pediatric plunging ranula. Int J Pediatr Otorhinolaryngol. 2006 Jun;70(6):1049-54. doi: 10.1016/j.ijporl.2005.10.022. Epub 2005 Dec 13. PMID: 16356556. | Study without a comparison group. |
| McClatchey KD, Appelblatt NH, Zarbo RJ, Merrel DM. Plunging ranula. Oral Surg Oral Med Oral Pathol. 1984 Apr;57(4):408-12. doi: 10.1016/0030-4220(84)90160-9. PMID: 6584837. | Study without follow-up. |
| McGurk M, Eyeson J, Thomas B, Harrison JD. Conservative treatment of oral ranula by excision with minimal excision of the sublingual gland: histological support for a traumatic etiology. J Oral Maxillofac Surg. 2008 Oct;66(10):2050-7. doi: 10.1016/j.joms.2008.01.019. PMID: 18848101. | Study without a comparison group. |
| Mizuno A, Yamaguchi K. The plunging ranula. Int J Oral Maxillofac Surg. 1993 Apr;22(2):113-115. | Study without follow-up. |
| Morton RP, Ahmad Z, Jain P. Plunging ranula: congenital or acquired? Otolaryngol Head Neck Surg. 2010 Jan;142(1):104-7. doi: 10.1016/j.otohns.2009.10.014. PMID: 20096232. | Study without a comparison group. |
| Nguyen BN, Malone BN, Sidman JD, Barnett Roby B. Excision of sublingual gland as treatment for ranulas in pediatric patients. Int J Pediatr Otorhinolaryngol. 2017 Jun;97:154-156. doi: 10.1016/j.ijporl.2017.04.003. Epub 2017 Apr 5. PMID: 28483227. | The study did not report the type of ranula/ surgical treatments. |
| O'Connor R, McGurk M. The plunging ranula: diagnostic difficulties and a less invasive approach to treatment. Int J Oral Maxillofac Surg. 2013 Nov;42(11):1469-74. doi: 10.1016/j.ijom.2013.03.019. Epub 2013 May 29. PMID: 23726274. | Study without a comparison group. |
| Oloyede OA. Prenatal management of choroid plexus cyst in a developing country: case report. J Obstet Gynaecol. 2020 Feb;40(2):273-274. doi: 10.1080/01443615.2019.1623181. Epub 2019 Jul 29. PMID: 31352851. | Study without a comparison group. |
| Pandit RT, Park AH. Management of pediatric ranula. Otolaryngol Head Neck Surg. 2002 Jul;127(1):115-8. doi: 10.1067/mhn.2002.126590. PMID: 12161740. | Study without a comparison group. |
| Pérez-de-Oliveira ME, Durighetto Junior AF, Ramos-Perez FM, da Cruz Perez DE. Micromarsupialization: A minimally invasive alternative for treatment of a large oral ranula. J Craniofac Surg. 2016 Oct;27(7):1919-1920. doi: 10.1097/SCS.0000000000003056. | Study without a comparison group. |
| Plant RL, Kokesh JF, Zwack GC. 10:48 AM: A Comparison of Oral and Transcervical Removal of Ranulas. *Otolaryngology–Head and Neck Surgery*. 2006;135(2_suppl):P209-P209. doi:[10.1016/j.otohns.2006.06.1014](https://doi.org/10.1016/j.otohns.2006.06.1014) | Abstract. |
| Raju R, Digoy GP. Management of the pediatric ranula. Oper Tech Otolaryngol Head Neck Surg. 2009 Dec;20(4):260-262. doi: 10.1016/j.otot.2009.09.003. | Review paper. |
| Ramalingaiah M, Nathan S. An analysis of twenty-five cases of ranula. J Evol Med Dent Sci. 2017;6(91):6476-6479. | Study without a comparison group. |
| Sandrini FA, Sant'ana-Filho M, Rados PV. Ranula management: suggested modifications in the micro-marsupialization technique. J Oral Maxillofac Surg. 2007 Jul;65(7):1436-8. doi: 10.1016/j.joms.2006.06.291. PMID: 17577520. | Study without a comparison group. |
| Seo JH, Park JJ, Kim HY, Jeon SY, Kim JP, Ahn SK, Hur DG, Kim DW, Lee JS. Surgical management of intraoral ranulas in children: an analysis of 17 pediatric cases. Int J Pediatr Otorhinolaryngol. 2010 Feb;74(2):202-5. doi: 10.1016/j.ijporl.2009.11.011. Epub 2009 Dec 14. PMID: 20005579. | Study without a comparison group. |
| Sigismund PE, Bozzato A, Schumann M, Koch M, Iro H, Zenk J. Management of ranula: 9 years' clinical experience in pediatric and adult patients. J Oral Maxillofac Surg. 2013 Mar;71(3):538-44. doi: 10.1016/j.joms.2012.07.042. Epub 2012 Sep 23. PMID: 23010374. | Study without a comparison group. |
| Ranula management by modified micromarsupialization technique in elderly patient. Oral Surg Oral Med Oral Pathol Oral Radiol. 2014 Feb;117(2):e187-e188. doi: 10.1016/j.oooo.2013.12.198. | Abstract. |
| Studart Soares EC, de Medeiros JR, Costa FWG, Bezerra TP, Nogueira CBP, de Freitas Silva MR, Sousa FB. Ránulas intraorales en una población brasileña: estudio retrospectivo del empleo de la marsupialización modificada. Acta Odontol Venez. 2012;50(3). | Study without follow-up. |
| Torres Y, Brygo A, Ferri J. A 17-year surgical experience of the intraoral approach for ranulas. J Stomatol Oral Maxillofac Surg. 2018 Jun;119(3):172-176. doi: 10.1016/j.jormas.2018.02.011. Epub 2018 Mar 1. PMID: 29501804. | Study without follow-up. |
| de Visscher JG, van der Wal KG, de Vogel PL. The plunging ranula. Pathogenesis, diagnosis and management. J Craniomaxillofac Surg. 1989 May;17(4):182-5. doi: 10.1016/s1010-5182(89)80020-4. PMID: 2659625. | Study without a comparison group. |
| Yang Y, Hong K. Surgical results of the intraoral approach for plunging ranula. Acta Otolaryngol. 2014 Feb;134(2):201-5. doi: 10.3109/00016489.2013.831481. Epub 2013 Oct 16. PMID: 24128284. | Study without a comparison group. |
| Woo SH, Chi JH, Kim BH, Kwon SK. Treatment of intraoral ranulas with micromarsupialization: clinical outcomes and safety from a phase II clinical trial. Head Neck. 2015 Feb;37(2):197-201. doi: 10.1002/hed.23579. Epub 2014 Mar 21. PMID: 24375530. | Study without a comparison group. |
| Yoshimura Y, Obara S, Kondoh T, Naitoh S. A comparison of three methods used for treatment of ranula. J Oral Maxillofac Surg. 1995 Mar;53(3):280-2; discussion 283. doi: 10.1016/0278-2391(95)90224-4. PMID: 7861278. | Study without a comparison group. |
| Yin T, Jain P, Ahmad Z, Harrison JD, Morton RP. Bilateral Plunging Ranulas in South Auckland: Evidence for a Genetic Basis. Laryngoscope. 2021 Jan;131(1):73-77. doi: 10.1002/lary.28593. Epub 2020 Feb 28. PMID: 32109322. | The study did not report the type of ranula/ surgical treatments. |
| Zhang L, McGurk M, Thomas B, Harrison JD. Rationale in the management of salivary gland ranula. Int J Oral Maxillofac Surg. 2009 May;38(5):575. | Abstract. |
| Zhao YF, Jia Y, Chen XM, Zhang WF. Clinical review of 580 ranulas. Oral Surg Oral Med Oral Pathol Oral Radiol Endod. 2004 Sep;98(3):281-7. doi: 10.1016/S1079210404000800. PMID: 15356464. | The study did not report the type of ranula/ surgical treatments. |
| Zhao YF, Jia J, Jia Y. Complications associated with surgical management of ranulas. J Oral Maxillofac Surg. 2005 Jan;63(1):51-4. doi: 10.1016/j.joms.2004.02.018. PMID: 15635557. | The study did not report the type of ranula/ surgical treatments. |
| Zhao Q, Li M, Lai R, Wang S. Treatment of intraoral ranulas with a two-incision fistula technique: the management of recurrence. Br J Oral Maxillofac Surg. 2018 Feb;56(2):129-133. doi: 10.1016/j.bjoms.2017.12.013. Epub 2018 Jan 12. PMID: 29338892. | Study without a comparison group. |
| Zhi K, Wen Y, Ren W, Zhang Y. Management of infant ranula. Int J Pediatr Otorhinolaryngol. 2008 Jun;72(6):823-6. doi: 10.1016/j.ijporl.2008.02.012. Epub 2008 Apr 2. PMID: 18387677. | Duplicate study. |
| Zhi K, Wen Y, Zhou H. Management of the pediatric plunging ranula: results of 15 years' clinical experience. Oral Surg Oral Med Oral Pathol Oral Radiol Endod. 2009 Apr;107(4):499-502. doi: 10.1016/j.tripleo.2008.09.023. Epub 2008 Dec 13. PMID: 19071033. | Duplicate study. |

**Appendix Figure 1:** Direct estimates for recurrence – oral ranula.


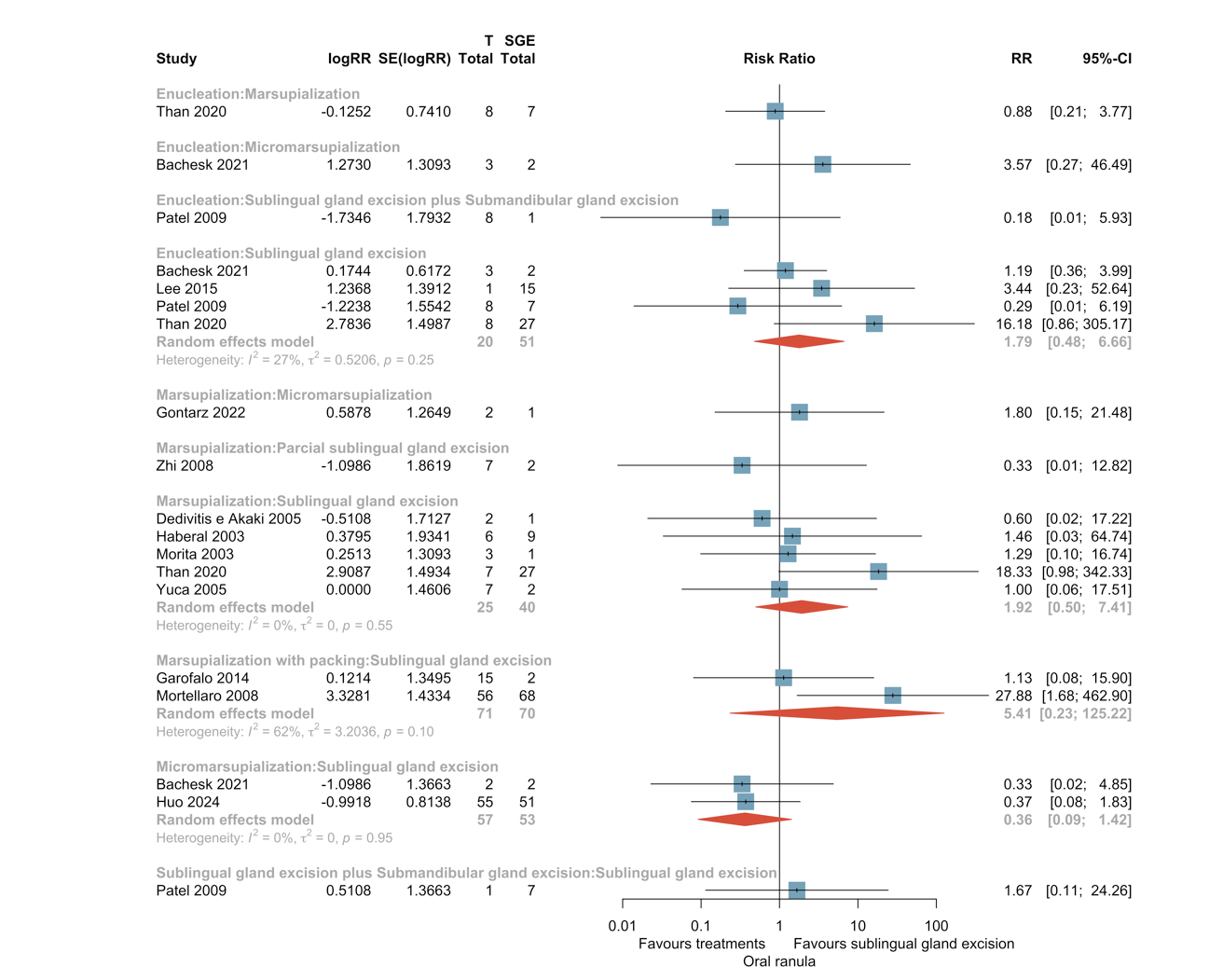


**Appendix Figure 2:** Direct estimates for recurrence – plunging ranula.

**
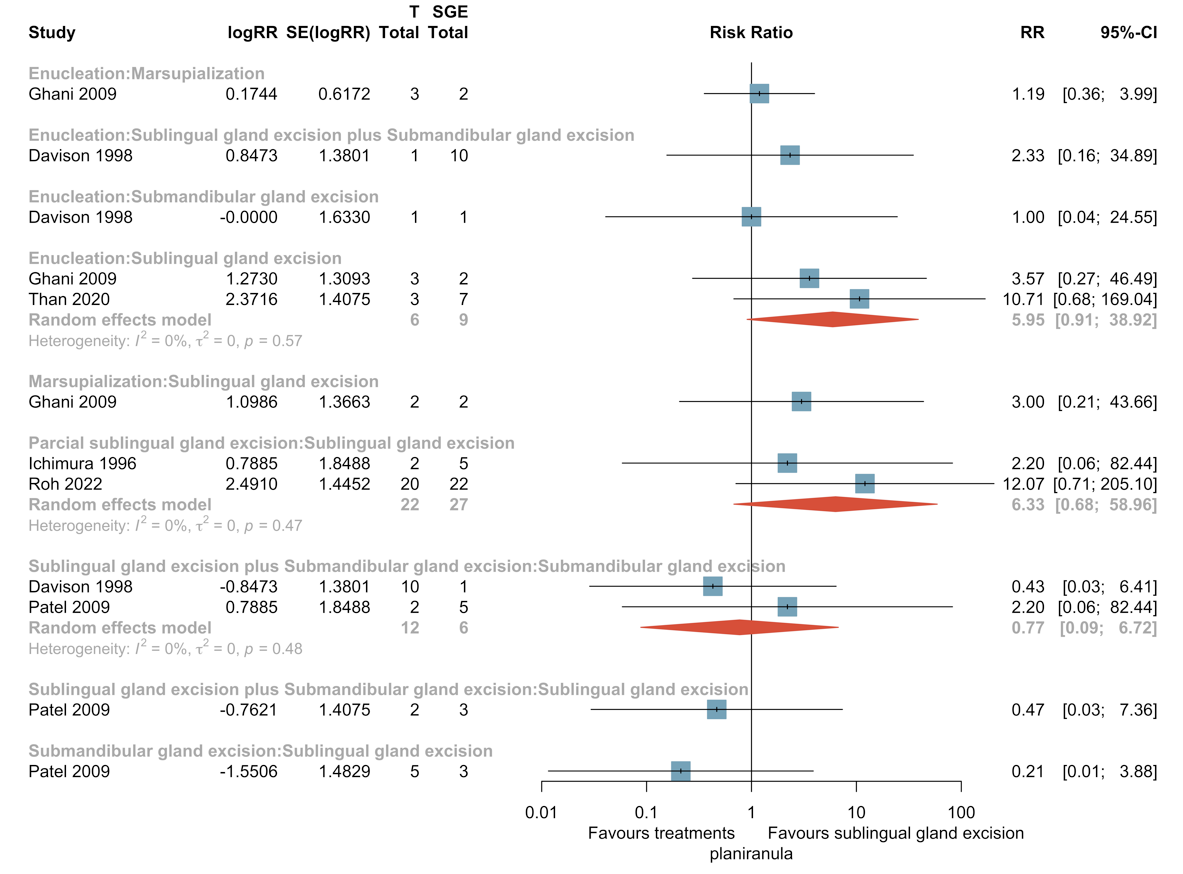
**

**Appendix Figure 3:** Network estimates for recurrence – oral ranula.

**
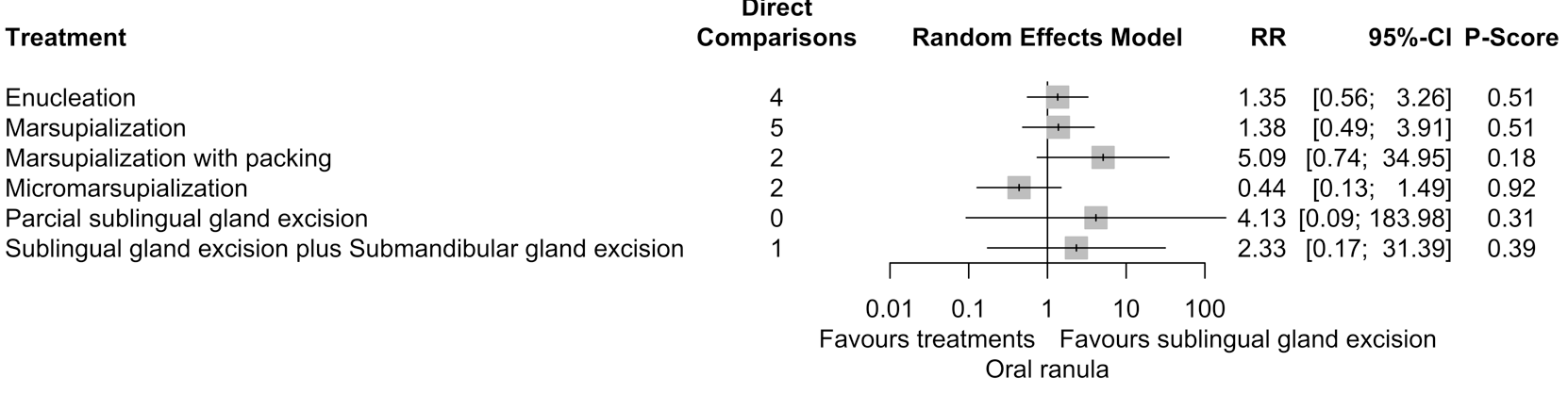
**

**Appendix Figure 4:** Network estimates for recurrence – plunging ranula.

**
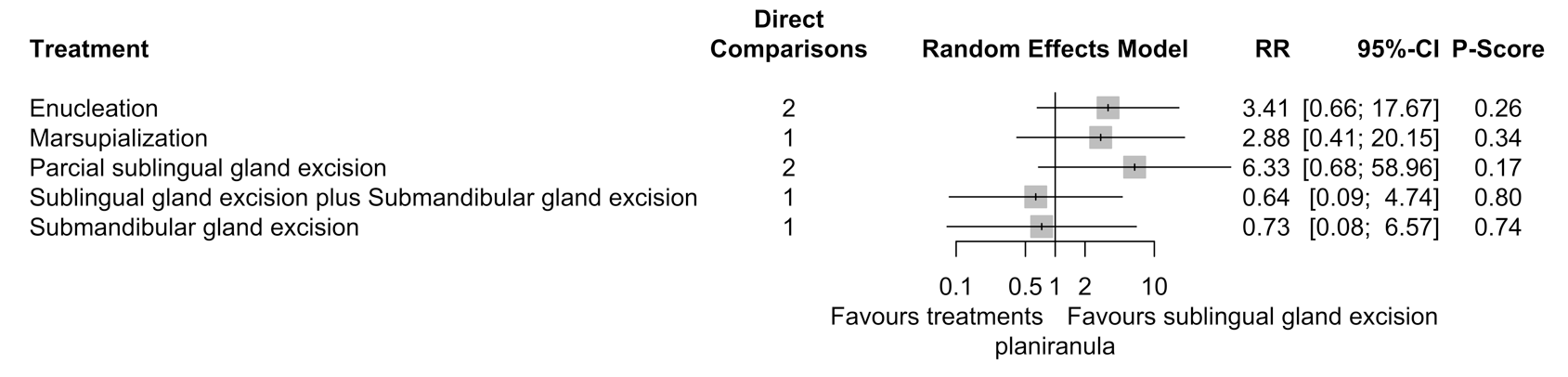
**

**References cited in the Appendix**

[1] Bonner A, Alexander PE, Brignardello-Petersen R, Furukawa TA, Siemieniuk RA, Zhang Y, et al. Applying GRADE to a network meta-analysis of antidepressants led to more conservative conclusions. J Clin Epidemiol 2018;102:87–98. https://doi.org/10.1016/j.jclinepi.2018.05.009.

[2] Guyatt G, Oxman AD, Akl EA, Kunz R, Vist G, Brozek J, et al. GRADE guidelines: 1. Introduction—GRADE evidence profiles and summary of findings tables. J Clin Epidemiol 2011;64:383–94. https://doi.org/10.1016/j.jclinepi.2010.04.026.

[3] Higgins JPT, Thomas J, Chandler J, Cumpston M, Li T, Page MJ, et al. Cochrane Handbook for Systematic Reviews of Interventions, version 6.5, Chapter 10 (updated August 2024). Cochrane, 2024. Available from: www.training.cochrane.org/handbook.; 2024.

[4] Brignardello-Petersen R, Bonner A, Alexander PE, Siemieniuk RA, Furukawa TA, Rochwerg B, et al. Advances in the GRADE approach to rate the certainty in estimates from a network meta-analysis. J Clin Epidemiol 2018;93:36–44. https://doi.org/10.1016/j.jclinepi.2017.10.005.

[5] Puhan MA, Schunemann HJ, Murad MH, Li T, Brignardello-Petersen R, Singh JA, et al. A GRADE Working Group approach for rating the quality of treatment effect estimates from network meta-analysis. BMJ 2014;349:g5630–g5630. https://doi.org/10.1136/bmj.g5630.

[6] Lu G, Ades AE. Assessing Evidence Inconsistency in Mixed Treatment Comparisons. J Am Stat Assoc 2006;101:447–59. https://doi.org/10.1198/016214505000001302.

[7] Brignardello-Petersen R, Johnston BC, Jadad AR, Tomlinson G. Using decision thresholds for ranking treatments in network meta-analysis results in more informative rankings. J Clin Epidemiol 2018;98:62–9. https://doi.org/10.1016/j.jclinepi.2018.02.008.
